# Flexural behaviour and evaluation of ultra-high-performance fibre reinforced concrete beams cured at room temperature

**DOI:** 10.1038/s41598-021-98502-x

**Published:** 2021-09-24

**Authors:** Jinlin Huang, Zhiying He, Muhammad Basit Ehsan Khan, Xiaohong Zheng, Zhibin Luo

**Affiliations:** 1 School of Water Conservancy and Civil Engineering, South China Agriculture University, Guangzhou, 510642 China; 2 School of Civil Engineering, The University of Sydney, Sydney, NSW 2006 Australia; 3 School of Civil Engineering and Transportation, South China University of Technology, Guangzhou, 510640 China; 4 Department of Civil Engineering, Pakistan Institute of Engineering and Technology, Multan, 66000 Pakistan

**Keywords:** Engineering, Materials science

## Abstract

Heat treatment is often required for ultra-high-performance concrete (UHPC) to achieve high strength. To broad its use in construction, the effect of different curing conditions on the properties of UHPC has been developed for many years. The experimental investigation of large scale ultra-high-performance fibre reinforced concrete (UHPFRC) beams is limited. In the present study, UHPFRC specimens and concrete cured at 20 °C were prepared to investigate the properties and flexural behaviour. The standard cubic compressive strength of UHPFRC specimens cannot be achieved at curing temperature of 20 °C. The bearing capacity under flexure was enhanced with the increase of reinforcement ratio. The failure modes of beams changed from ductile to brittle as the reinforcement ratio increased from 1.26 to 9.50%. The flexural behaviour of UHPFRC beams cured at room temperature was in accordance with the UHPFRC beams cured at high temperature in previous studies. In addition, the calculation model of CECS38-2004 underestimated the bending moment capacity of the under-reinforced UHPFRC beams (with reinforcement ratio from 0 to 7.85%) and overestimated the bending moment capacity of the UHPFRC beams with high reinforcement ration of 9.50%.

## Introduction

Ultra-high performance fibre reinforced concrete (UHPFRC) is a form of concrete with a ultra-high compressive strength (150 to 200 MPa), a high tensile strength (> 7 MPa), a high bending strength and a low water-cement ratio of 0.2 or less^1^. It has been an attractive choice for high buildings and long span bridges due to its superior mechanical performance. The raw materials of UHPFRC consist of cement, fine sand, silica fume, quartz powder, superplasticizer, fibres and the mineral additives with different binding characteristics^2,3^. Many studies have been performed to investigate the properties and mechanical behaviour of UHPFRC. The mechanical behaviour and strength of UHPFRC depend on the quality of raw materials, the mix proportion and the curing regimes. The effect of mixture ratios on the strength of UHPFRC has been examined at a wide range. The water-binder and water-cement ratios maintained at approximately 0.16 to 0.20 in UHPFRC^4,5^. The compressive strength of UHPFRC increased with the decrease of the ratio of water to cement because of the enhancing interface area of aggregate-matrix and fibre-matrix^5^. The high range of water reducer was employed to retain the workability of UHPFRC with low water-cement ratio^6,7^.

The concrete could obtain excellent mechanical properties by adding fibres. The fibres in UHPFRC supplied a crack control and resistance to the pull-out force for the cement matrix after failure^5^. Thus, a number of research focus on the effects of fibre including the geometry^8^, length^9,10^ and volume fractions^11^, the orientation^12,13^ and the type^14^. With a constant cross-sectional area, the triangular and square-shape fibres were more efficient in improving the mechanical bond than the circular-shaped^15^. Moreover, the UHPFRCs with the twisted addition of hooked at end steel fibres had a greater tensile strength than those with short straight-line fiber of steel^16^. The flexural resistance, deflection ability and robustness of UHPFRC were improved by increasing the fibre length^10^. However, the UHPFRC with shorter fibres had greater workability^5^. Moreover, for a constant fibre volume fraction, the shorter the length of the fibre, the greater the amount of fibres on the crack area, which affecting the tensile behaviour of the UHPFRC.

The properties of UHPFRC also depend on the curing conditions^17^. Autoclaving at a moderate temperature of 65 °C and cured at around 85 to 90 °C are employed in UHPFRC. The UHPFRC showed higher compressive strength after steam and autoclave curing at 90 °C than that under room temperature curing^18–20^. The improvement in the compressive strength under high temperature is due to the accelerated hydration of cementitious material and the secondary hydration between mineral admixtures and calcium hydroxide^21^. Moreover, higher curing temperature showed positive effect on the modulus of elasticity of UHPC due to enhanced hydration of cementitious materials^22^. The flexural strength of UHPFRC produced with ground granulated blast-furnace slag (GGBS) was significantly improved under higher temperature curing. The increase in flexural strength can be attributed to bonds between matrix and aggregate which are improved by the increasing rate of reaction of binder and CSH chain length under high temperature curing^19^. Moreover, the porosity and density of UHPFRC can be reduced when the specimens were cured at high temperature due to expansion of CSH gel^3^.

However, the high temperature and long duration curing for UHPFRC limit its broader application due to the high energy consumption and manufacturing price and the low production efficiency. Developing UHPFRC without heat treatment would promote the widespread use of the product. Thus, many extensive studies of UHPFRC cured at room temperature have been carried out^18,19,23–26^. Table 1 summarizes the test results of previous studies of the UHPFRC cured at room temperature. These studies investigated and acquired the mechanical properties of the UHPFRC such as the compressive strength and tensile strength. However, the flexural behaviour of large scale UHPFRC beams have not been studied in these studies. A number of research have investigated the effect of raw materials, mix proportion, fibres and curing conditions on the flexural behaviour of large scale UHPFRC beams^27–33^. To the best of authors’ knowledge, the investigation of flexural behaviour of UHPFRC beams cured at room temperature is limit. Some research studies have demonstrated the flexural behaviour of UHPFRC beams cured at room temperature^27,33^ but only a small range of reinforcement ratio was employed. Importantly, the load carrying capacity, failure modes, and ductility depend on the reinforcement ratio of concrete beams to some extent^34,35^. Concrete beams could obtain higher loading capacity with an increase reinforcement ratio^34^ while higher ductility (ductile failure) had been observed in low reinforcement ratio^35^. Thus, comprehensive understanding of the mechanical behaviour and properties of UHPFRCs with different reinforcement ratios cured at room temperature are still required.

**Table 1:** Tests results of UHPFRCs cured at room temperature.

References	Dimensions	Test types	Results MPa
^18^	Prismatic specimens (40 × 40 × 160 mm^3^)	Flexural strength	23
	Cylindrical specimens (100 × 200 mm^3^)	Compressive strength	163
^19^	Prismatic specimens (40 × 40 × 160 mm^3^)	Flexural strength	27.8
^25^	Cubic specimens (50 × 50 × 50 mm^3^)	Compressive strength	110
^26^	Cubic specimens (50 × 50 × 50 mm^3^)	Compressive strength	125
	Prismatic specimens (50 × 50 × 200 mm^3^)	Flexural strength	22
^36^	Cylindrical specimens (R = 50 mm H = 100 mm)	Compressive strength	162
	Plate specimens (100 × 12 × 400 mm^3^)	Flexural strength	35
	Beam specimens (100 × 100 × 400 mm3)	Tensile strength	23
^37^	Prismatic specimens (50 × 200 × 500 mm^3^)	Compressive strength	168
		Tensile strength	11

The aim of this paper is to examine the flexural behaviour of the UHPFRC beams with a wide range of reinforcement ratio from 0 to 9.5% cured at room temperature. The investigated behaviours included crack patterns, failure mode, flexural behaviour, and ductility. Basic mechanical properties such as compressive strength, tensile strength and flexural strength of UHPFRC specimens were determined prior to the investigation of UHPFRC beams. Experimental results of the UHPFRC beams were compared to the theoretical prediction by the Chinese standards to evaluate the applicability of these standards in UHPFRC cured at room temperature. Following Codes were used in this study: “Technical specification for fibre reinforced concrete structures” CECS38-2004^38^, “Standard for test method of mechanical properties on ordinary concrete” GB/T 50081-2019^39^, “Technical Specification for Reactive Powder Concrete Structures” DBJ43T325-2017^40^.

## Experimental program

Experimental program involved characterizing the material properties and determining the parameters of the calculation model and included studying the flexural behaviour of UHPFRC beams.

### Materials and mixture proportion

**Figure 1:**
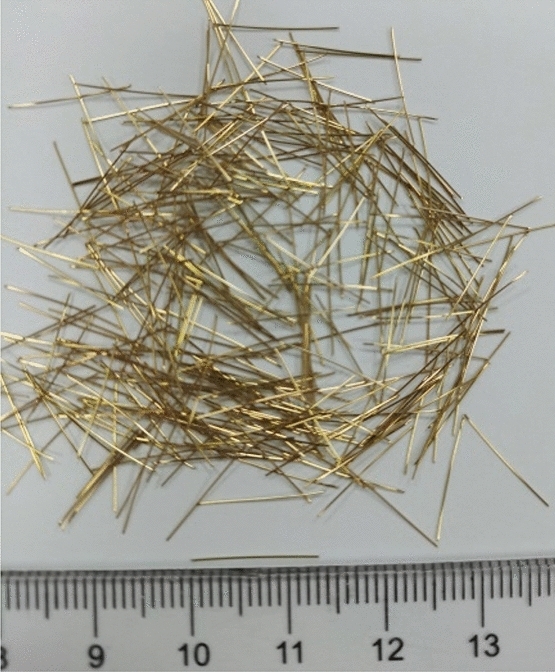
The steel fibres.

An ultra-high-performance fibre-reinforced cementitious composite was prepared in this study. The range of mixture ratios to acquire excellent UHPFRC were examined and summarized by many research studies. In order to manufacture UHPFRC specimens with high quality, the mixture proportions were chosen from the tests database^5^. Preferably, the water-cement ratio maintained at 0.16 to 0.20. The silica fume and silica flour of 10% to 30% of the cement mass were required to fill the voids between cement particles. The proportions of aggregate and superplasticizer were 110% and 2% of the cement mass, respectively. Moreover, the steel fibres content was recommended as a volume fraction of 2% to 3% based on the workability and mechanical performance of UHPFRC. The mixture proportions used in this study are summarized in Table 2. The cement used in this study was Ordinary Portland cement 42.5R. Silica sand with diameter of 360 to 600 μm and 600 to 840 μm was used as aggregates. Silica fume including 94% SiO_2_ with a diameter of 0.1 μm and silica flour with a diameter of 50 μm were added as fillers. The Dramix steel fibres were added at 2% by volume of the entire mix. As shown in Fig. 1, the steel fibres had 13 mm length and 0.2 mm diameter with a yield strength of 2850 MPa as reported by the manufacturer.

**Table 2:** Proportion of materials in the UHPFRC mixture by weight ratio (SSA = silica sand with diameter (360–600 μm), SSB = silica sand with diameter (600–840 μm), and SP = superplasticizer).

Cement	Water	SSA	SSB	Silica flour	Silica fume	SP	Steel fibre
1.0	0.18	0.30	0.80	0.30	0.30	0.02	2% by vol

#### UHPFRC mix design

The mixing procedure consisted of mixing the cement, silica fume, silica flour and sand in dry state for 2 min. Then, the water mixed with superplasticizer was added into the dry mixture and mixed for 6 min. When the mixture became visibly flowable, the steel fibres were added and mixed for further 5 min.

### Specimens and test methods

#### Compressive, tensile and flexural strength tests

**Figure 2:**
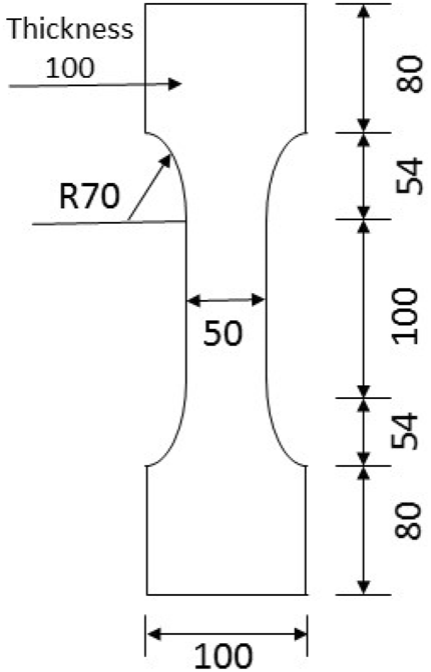
Dimensions of dog-bone shaped specimen.

A series compression and tension tests were conducted to characterize the material properties. To identify the compressive strength, specimens with dimensions of 100 × 100 × 100 mm^3^ and 100 × 100 × 300 mm^3^ were casted and tested. Dog-bone shaped specimens with a rectangular cross section of 50 mm × 100 mm and length of 368 mm were fabricated and tested for axial tensile strength as shown in Fig. 2. Specimens with dimension of 100 × 100 × 400 mm^3^ were tested for flexural tensile strength. All test specimens were reinforced with steel fibres but without rebars. These specimens were covered with plastic sheets immediately after casting and demoulded after 24 h. To identify the effect of curing temperature on the properties of UHPFRC, specimens with dimension of 100 × 100 × 100 mm^3^ were cured at three conditions including room temperature of 20 °C  ± 0.5 °C and steam curing at 60 °C  ± 0.5 °C and 90 °C  ± 0.5 °C for 48 h after demoulded. Specimens were then cured in a fog room at room temperature for 28 days and then tested. Specimens with dimensions of 100 × 100 × 300 mm^3^ and 100 × 100 × 400 mm^3^ were cured in a fog room at room temperature for 28 days after demoulded and then tested. The compressive strength, tensile strength and flexural strength for each group were determined with three specimens.

### Large scale beam specimens

**Figure 3:**
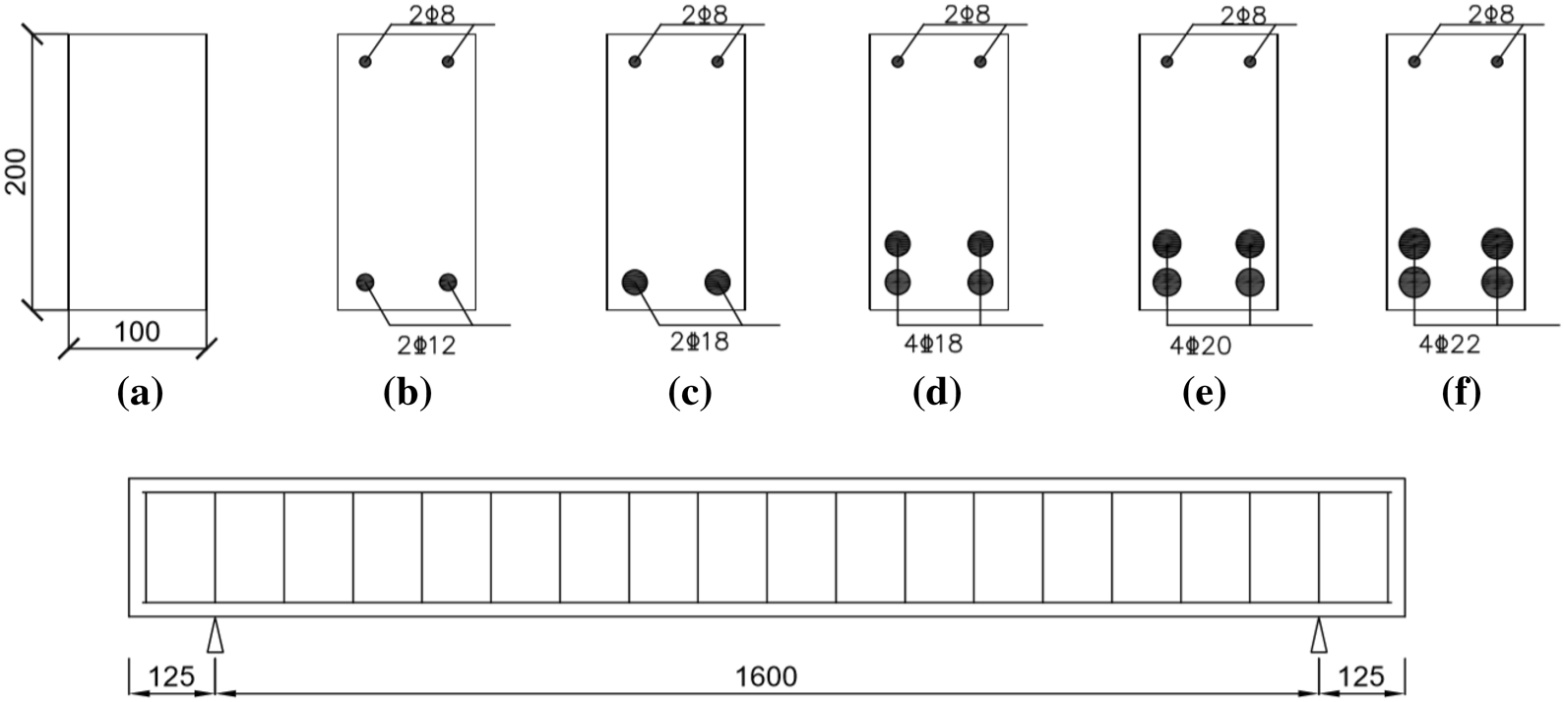
Cross section and spans detail of concrete beams.

Six groups of beams with different reinforcement ratios were fabricated and tested in this study as shown in Fig. 3. Each group consisted of three specimens. Control beams without any reinforcement were labelled as NR. For reinforced beams, rebars at the top were kept same i.e., single layer of two bars with 8 mm diameter. The beams were designated according to the arrangement of bottom reinforcement rebars. Beams with one rebar layer at the bottom with two 12 mm rebars were labelled as R12-1. R18-1 had one rebar layer at the bottom with two bars of 18 mm diameter. R18-2 had two layers of reinforcement at the bottom with two rebars of 18 mm in each layer while R20-2 had two reinforcement layers at the bottom containing two rebars of 20 mm per layer. R22-2 had two layers of rebars with two bars of 22 mm in each layer. All beams had the similar dimensions with an effective span length of 1600 mm and cross-section of 100 mm × 200 mm. To prevent any premature shear failure of the beam, shear reinforcement was provided. The shear reinforcement consisted of stirrups of 8 mm diameter spaced at 100 mm centre to centre throughout the beam.

The beams were fabricated one at a time because of the concrete mixer of 1000 L capacity and the larger quantity of mixtures required. The beams were fabricated by placing the concrete using back and forth placement method along the span of the beam. The specimens were covered with wet hessian and plastic sheets immediately after concrete casting and cured at room temperature for the first 24 h, prior to demolding. After demolding, the specimens were cured in a fog room at room temperature of 20 °C ± 0.5 °C for 28 days.

### Test setup

#### Compressive, tensile and flexural strength tests setup

**Figure 4:**
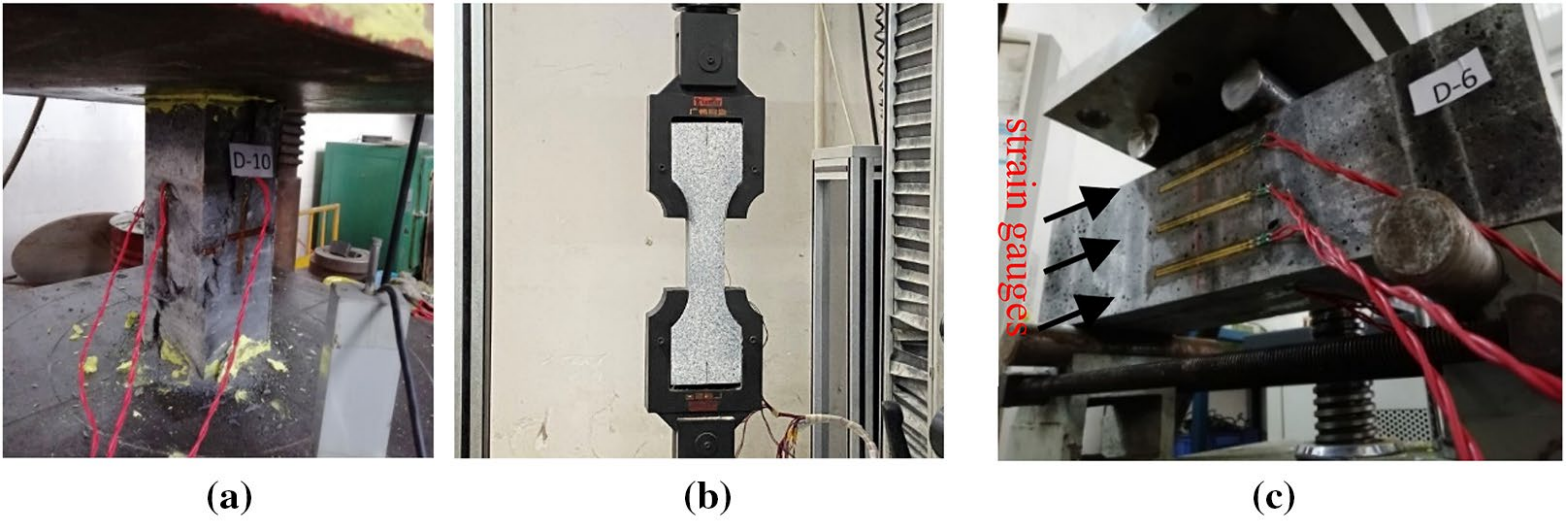
(**a**) Compressive strength test; (**b**) Tensile strength test; (**c**) Flexural strength test.

The compressive strength and flexural strength of the steel fibre-reinforced concrete were determined based on GB50081-2019. Specimens with dimension of 100 × 100 × 100 mm^3^ and 100 × 100 × 300 mm^3^ were used to determine the compressive strength by uniaxial compressive load applied at rate of 0.8 to 1.0 MPa/s. For the specimens with dimension of 100 × 100 × 300 mm^3^, the electrical strain gauges of 80.1 mm length were attached at the mid height to record the axial and lateral strains as show in Fig. 4a. Additionally, a dial gauge was used to measure the platen-to-platen displacement to measure the strains of the whole specimen to determine the elastic modulus.

To determine the tensile strength, as shown in Fig. 4b, the dog-bone shaped specimens with a rectangular cross section of 50 mm × 100 mm and length of 368 mm were loaded at rate of 0.05 mm/min according to^41^. The strain of specimens was measured by digital image correlation (DIC). DIC is a reliable non-destructive method based on speckle tracking and is used to analyse the full displacement field on the surface of specimens by digital images^42–44^.

To determine the flexural strength, specimens with a dimension of 100 × 100 × 400 mm^3^ were loaded at rate of 0.08 to 0.1 MPa/s according to GB50081-2019. Three electrical strain gauges with a length of 80.1 mm were glued on the side surface of the specimen at midspan at different heights to measure the strain as shown in Fig. 4c.

#### Large scale beam test setup

**Figure 5:**
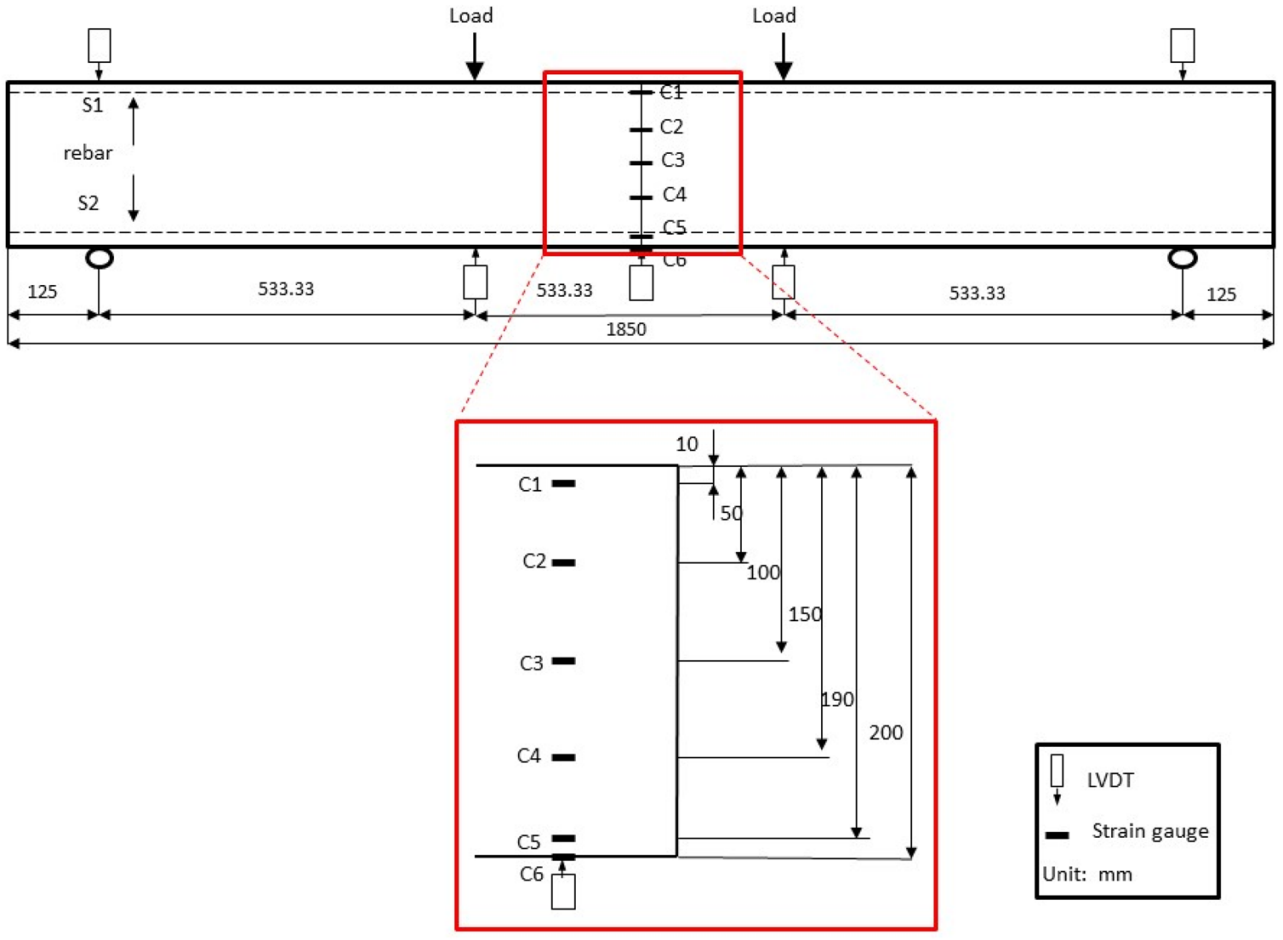
Schematic of the test-setup and strain gauges distribution of concrete beams.

The beams were subjected to two equally concentrated loads applied at 267 mm from the mid span via four-point loading method according to GB/T50081-2019. Figure 5 shows the loading configuration details of concrete beams. The beam was set up on a steel frame with a capacity of 1000 kN. A single hydraulically actuated jack was used to supply the monotonically increasing load. Load was supplied through displacement control method at the rate of 10 mm/min. As shown in Fig. 5, LVDTs and electrical resistance strain gauges were used to measure the deflections and strains of the beams. Five gauges with a length of 80.1 mm were glued on the side surface of the beam at midspan at different heights and two gauges were located on the bottom surface of the beam at midspan. In addition, electrical strain gauges with length of 1 mm were glued to the steel rebar at midspan before the casting of the beam to measure the strains of steel.

## Results and discussion

### Properties of the UHPFRC specimens

Table 3 shows the test results of the UHPFRC specimens. It can be seen that the average cubic compressive strength increases from 102.90 to 156.46 MPa with the increase of curing temperature from 20 to 90 °C, which is consistent to the aforementioned literatures. The strength level of cubic compressive strength of UHPFRC was achieved for specimens cured at 90 °C, indicating that the mixture proportion and manufacturing method employed in this study can satisfy the strength requirement of normal UHPFRC^5^. The compressive strength of specimen with dimension of 100 × 100 × 300 mm^3^ was 79.08 MPa which was lower than the cubic compressive strength. It is stated that larger specimens released more stored energy than smaller specimens^5^.

**Table 3:** Summary of test results.

Dimension (mm^3^)	Curing temperature °C	Compressive strength MPa	Flexural strength MPa	Tensile strength MPa
100 × 100 × 100	20	102.90	–	–
	60	119.92		
	90	156.46		
100 × 100 × 300	20	79.08	–	–
100 × 100 × 400	20	–	17.7	–
Dog-bone shaped	20	–	–	8.38

The specimens with dimension of 100 × 100 × 400 mm^3^ were tested under four-point loading and the flexural strength was 17.7 MPa. The flexural strength of the specimens under four-point loading was calculated by Eq. (1) according to GB50081-2019 as follow:

1 $$ f_{f} = \frac{Fl}{{bh^{2} }} $$

where F is the applied load at failure, l is the length of span measured bearing to bearing, b is the width of cross-section and h is the depth of cross-section.

The axial tensile strength acquired from the dog-bone shaped specimen was 8.38 MPa.

### Test results for beams

#### Crack pattern and failure mode

**Figure 6:**
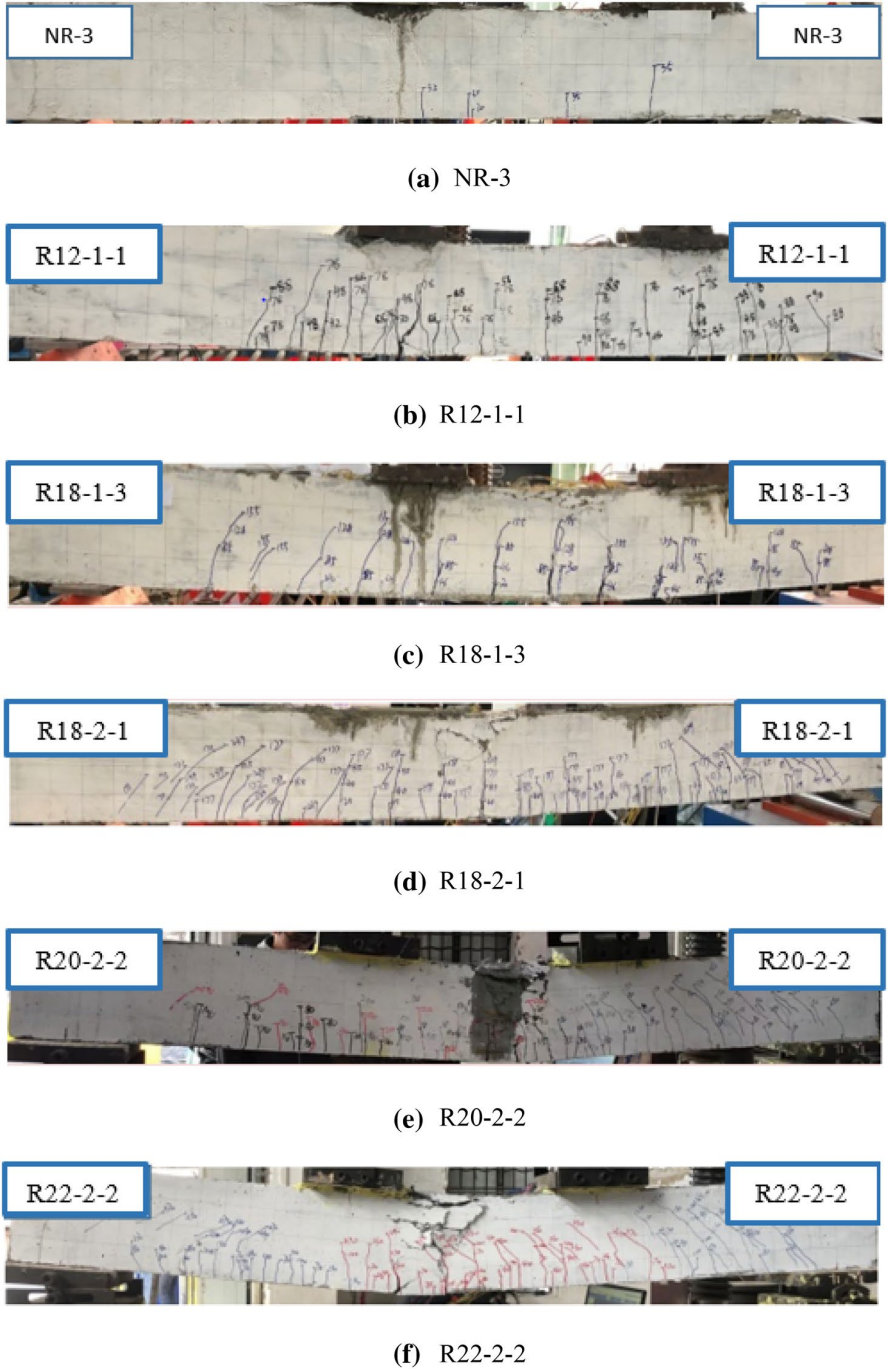
Modes of failure and crack patterns of the concrete beams.

For all the beams, the load increased linearly until the formation of first crack. The presence of the first cracks was audibly indicated followed by the first visible micro-cracks at the bottom surface of the beams between the loading points. The number of micro-cracks increased with the increase of the load, and new cracks propagated toward the upper face. One or two cracks in the middle portion of the beam became significantly visible whereas other cracks did not show any visible increase in width. In addition, the compression zone of concrete moved toward to the top as the cracks developing. As the test progressing, a noticeable increase in the number of cracks occurred and the steel fibres began to pull out. Given that steel fibres carried the tensile load and resisted the opening of the crack, the width of the cracks increased more rapidly with slight increase in load after the steel fibres began to pull out. The tensile load on the other nearby fibres increased, leading to pulling out of even more steel fibres. As shown in Fig. 6, the increase of reinforcement ratio increased the number of cracks and reduced the width of cracks.

Three to five visible cracks were observed for NR group beams between the loading points. The cracks of NR group beams propagated to the depth of 0.5 h (height of the beam) at failure. All R group beams exhibited vertical cracks. In addition to vertical cracks, the number of diagonal cracks in the region between the loading point and the support increased with the increase of reinforcement ratio. One or two major cracks were formed during failure and then developed rapidly in length and width. Moreover, the depth of the crack increased with the increase of reinforcement ratio from 1.26 to 7.85%. At the end of the tests, the depths of crack for R12-1, R18-1, and R18-2 were about 0.60 h, 0.70 h, and 0.75 h, respectively. The depths of the crack for R20-2 and R22-2 were about 0.85 h and 0.7 h, respectively.

Concrete is known as a brittle material and shows immediate loss of load carrying capacity without reinforcement at failure^45^. Brittle failure was observed in NR beams as the ordinary concrete. The failure modes of R group beams were different from those of NR group beams, depending on the longitudinal reinforcement ratio. In terms of reinforcement ratio, the beams can be divided in to three groups: under-reinforced beams, balanced-reinforced beams and over-reinforced beams. Failure of under-reinforced beams was gradual and was accompanied by fairly large deflection. The ultimate load capacity of the beams increased with an increase in tensile reinforcement ratio but the deflection ductility decreased, leading to brittle failure^34^. Thus, failure of over-reinforced beams was more abrupt. The balanced-reinforced beams behaved in an intermediate manner between those of under-reinforced and over-reinforced beams. Moreover, ductile failure was always observed in under-reinforced beams while brittle failure was observed in over-reinforced beams. The balanced-reinforced beams appeared to fail in a fairly brittle manner^35^. Ductile failure occurred in under-reinforced beams because the steel yields and the concrete crushes simultaneously, causing considerable deformation^34^. With an increase of reinforcement ratio, the loading capacity of beams increases and the load distribution on each steel decreases. The concrete was crushed without prior yielding of the steel (less deformation), leading to a rapid propagation of cracks and sudden failure of concrete beams^35^. Ductile failure was observed for the beams R12-1, R18-1 and R18-2 with large deflection. As shown in Fig. 6b, c, d, the compression zone of the concrete crushing is small at failure. Thus, the reinforcement yielded before the beams reach the ultimate limit state in flexure. The yielding of reinforcement produced a ductile failure for these beams because the tensile reinforcement ratios of these beams were under-reinforced^27^. As shown in Fig. 6e, f, brittle failure was observed for the beams R20-2 and R22-2, accompanying with large concrete crushing of compression zone. The brittle failure indicated that the beams R20-2 and R22-2 could be balanced-reinforced or over-reinforced beams.

#### Load–deflection relationship

The failure loads and deflections of the tested beams are summarized in Table 4. It is clear to see that the flexural capacity of UHPFRC beams increased as reinforcement ratio increased. This trend is similar to other investigation^27,29,32,46^.

**Table 4:** Summary of flexural test results for reinforced UHPFRC beams. (ρ represents reinforcement ratio, P represents the load, and ∆ represents the mid-span deflection).

Beam	ρ (%)	First cracking				Peak state			
		P (kN)	Mean (kN)	∆ (mm)	Mean (mm)	P (kN)	Mean (kN)	∆ (mm)	Mean (mm)
NR									
1	0	31	31	0.99	0.91	45	40	2.82	2.32
2		24		0.82		34		1.88	
3		39		0.92		41		2.26	
R12-1									
1	1.26	89	91	5.80	5.98	105	102	17.67	18.97
2		93		6.30		100		21.79	
3		90		5.85		100		17.46	
R18-1									
1	2.83	160	165	7.33	7.42	188	188	25.52	25.09
2		179		6.99		198		23.79	
3		157		7.94		179		25.97	
R18-2									
1	6.36	225	226	9.54	9.27	263	257	12.89	12.21
2		227		8.79		258		11.38	
3		226		9.49		251		12.37	
R20-2									
1	7.85	253	244	11.64	11.02	273	261	15.76	14.03
2		223		9.65		241		12.57	
3		256		11.76		269		13.75	
R22-2									
1	9.50	275	264	11.20	10.96	301	281	13.84	13.75
2		263		11.49		270		13.78	
3		254		10.19		271		13.63	

**Figure 7:**
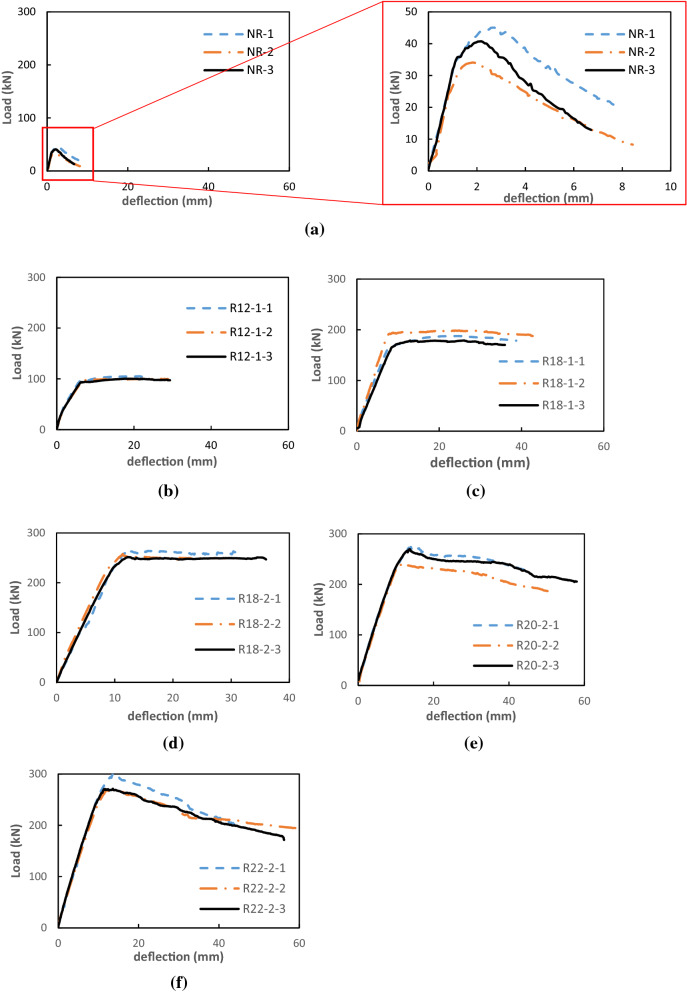
Load-mid-span deflection curves of concrete beams.

The deflection was measured at the mid span of the beam. Figure 7 shows the load–deflection relationship of the tested beams. Three distinct regions of the load–deflection relation can be observed in the NR group beams, including the linear zone before first cracking, yield stage, and rupture stage. The yield stage began when first cracking occurred and ended before reaching the maximum load. The rupture stage corresponded to that of strength losing. It can be seen that initial response of NR group beams was similar up to the peak load and then ruptured. The exact occurrence of the first crack was difficult to observe visually due to the multiple-cracking property of the UHPFRC. Thus, the crack load in this paper was defined as the load at the end of the initial linear stage in the load–deflection curve. The average peak load for the NR group beams was 40 kN and the average deflection measured for this load was 2.32 mm. After the peak was achieved, the specimens underwent into a softening stage, which showed a brittle failure pattern.

The beams R12-1, R18-1 and R18-2 showed similar trend in load–deflection behaviour. The deflection increased linearly and was proportional to the load until reaching the peak value of the load. After the peak value was achieved, the deflection increased while the load kept constant, showing a ductile failure pattern.

The load–deflection behaviour of the beams R20-2 and R22-2 was similar. The deflection increased linearly to the load until reaching its peak value. Then the load decreased progressively with the increase of the deflection.

#### Load-strain relationship

**Figure 8:**
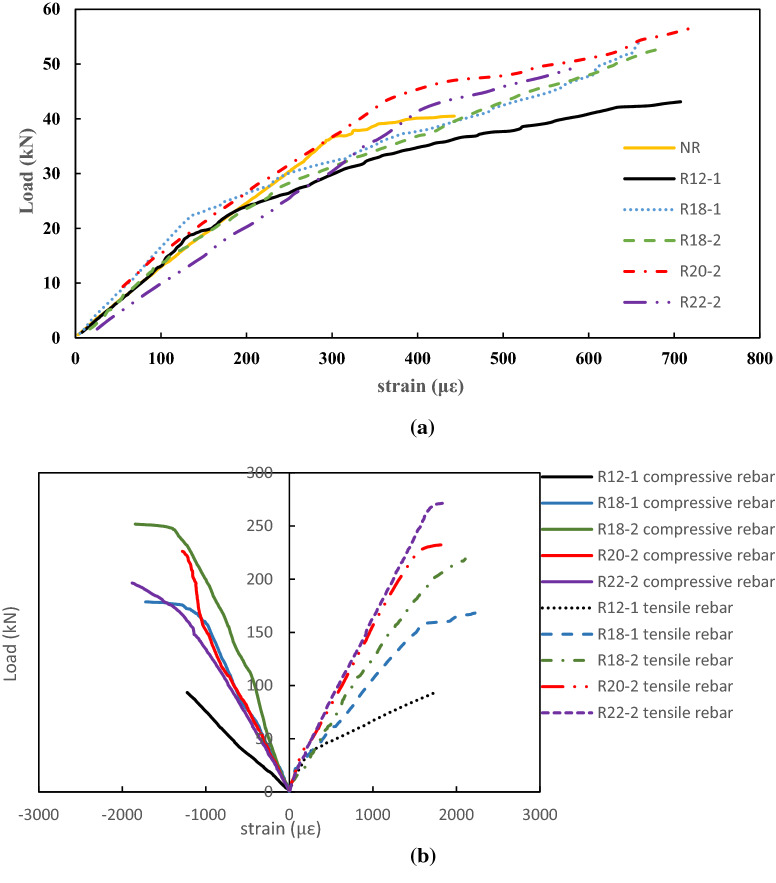
(**a**) Load-strain curves of the concrete at the bottom face. (**b**) Load-strain curves of the rebar.

The load-strain curves of the beams were shown in Fig. 8. The strain was measured by the strain gauges attached to the concrete and rebar surfaces. Negative strains represented compressive strains while positive strains represented tensile strains. As shown in Fig. 8a, the load-strain curves of the concrete at the bottom face elevated. The load-strain relationships were similar for all reinforcement ratios. The strain was linear at first followed by a nonlinear region. The nonlinear region started at the initiation of cracking. There is no abrupt change of strain at nonlinear region. The reason is that the steel fibres in the cement matrix resisted the tensile force after the initiation of cracking^32^. Unfortunately, most of the strain gauges attached to the bottom face of concrete came off due to the developing cracks. Thus, only load-strain relationship at strain from 0 to 700 με were shown in Fig. 8a.

Figure 8b shows that the strain of tensile rebars of all beams increased linearly at the beginning. Greater tensile strains were generated after the yielding of rebar occurred. It can be seen that the yielding point of tensile reinforcement increased as the reinforcement ratio increased. For the beams R12-1, R18-1 and R18-2, the tensile reinforcement yielded before the concrete crushed, indicating that the reinforcement ratio of these beams were under-reinforced. For the beams R20-2 and R22-2, as shown in Figs. 7 and 8, the load at the yield of tensile reinforcements closed to the load at the concrete crushed, indicating that the reinforcement ratios for R20-2 and R22-2 approached to balance-reinforced. The load-strain behaviour of tensile reinforcements is consistent to the failure modes of the concrete beams.

#### Ductility

The ductility of concrete beams can be quantified using the ductility index, which is expressed by the deflection ductility index, curvature ductility index or rotational ductility index^32^. Deflection of ductility index was adopted in this study as expressed in Eq. (2).

2 $$ \mu = \frac{{\Delta_{u} }}{{\Delta_{y} }} $$

where µ is the ductility index of the member, Δ_u_ is the mid-span deflection at the ultimate load, and Δ_y_ is the mid-span deflection at the yielding load. The ductility index for all the beams are shown in Table 5.

**Table 5:** Value of load–deflection behaviour (ρ represents reinforcement ratio, p_y_ represents the yielding load, p_u_ represents the ultimate load, and Ave represents the mean value, Stdev represents the standard deviation).

Beams			NR	R12-1	R18-1	R18-2	R20-2	R22-2
ρ (%)			0	1.26	2.83	6.36	7.85	9.50
Yielding state	p_y_ (kN)	Ave	27.97	91.58	166.07	225.38	251.06	265.08
		Stdev	2.93	1.98	9.60	5.03	14.71	10.25
	Δ_y_ (mm)	Ave	0.91	5.95	7.47	9.21	11.54	11.06
		Stdev	0.07	0.25	0.39	0.23	1.07	0.61
Ultimate state	p_u_ (kN)	Ave	39.94	101.59	187.91	255.88	260.93	280.81
		Stdev	4.53	2.37	8.00	3.70	14.52	13.96
	Δ_u_ (mm)	Ave	2.32	25.16	26.92	32.84	13.96	13.33
		Stdev	0.39	1.86	0.50	2.24	1.34	0.67
Ductility index	Δ_u_ /Δ_y_		2.55	4.23	3.60	3.57	1.21	1.21
	p_u_ /p_y_		1.43	1.11	1.13	1.14	1.04	1.06

It is well known that the natural brittleness of plain concrete disqualified it to be applied separately to the structure due to the requirement of ductility for safety. The addition of steel fibre increased the ductility of the NR beam to 2.55. The improvement of ductility by fibres has been discussed in previous studies^47–49^. The fibre reinforcement in a concrete mix can make complementary and additive contributions to tensile behaviour of the beam^49^. This is because the fibres induced the delay of macrocracks formation^48^. For the beams with rebar, the ductility decreased as the reinforcement ratio increased. The effect of the reinforcement ratio on the ductility of the beams is similar to previous studies^35,50,51^. This is because the tensile rebar yield before the concrete in the compression zone is crushed for low reinforcement ratio. As the reinforcement ratio increasing, the concrete will be crushed without prior yielding of tensile rebar, causing a brittle failure manner. Thus, the ductility of the beams decreased with the increase of reinforcement ratio^35^. However, the ductility indexes of beams R20-2 and R22-2 were less than that without rebar, indicating that the addition of steel fibre can improve the ductility of beams without reinforcement but weaken the ductility of beams with high reinforcement ratio. The reduced ductility index of beams with rebar by adding steel fibres was also reported in previous studies^52,53^. The reduction was caused by the smaller deflection capacity resulted from the crack bridging capability of fibres, leading to the decreases in differences between deflections at steel rebar yield and peak load^54^.

## Estimation of bending moment capacity

In China, the bending moment capacity of UHPFRC members is predicted by the calculation model in CECS38-2004.

**Figure 9:**
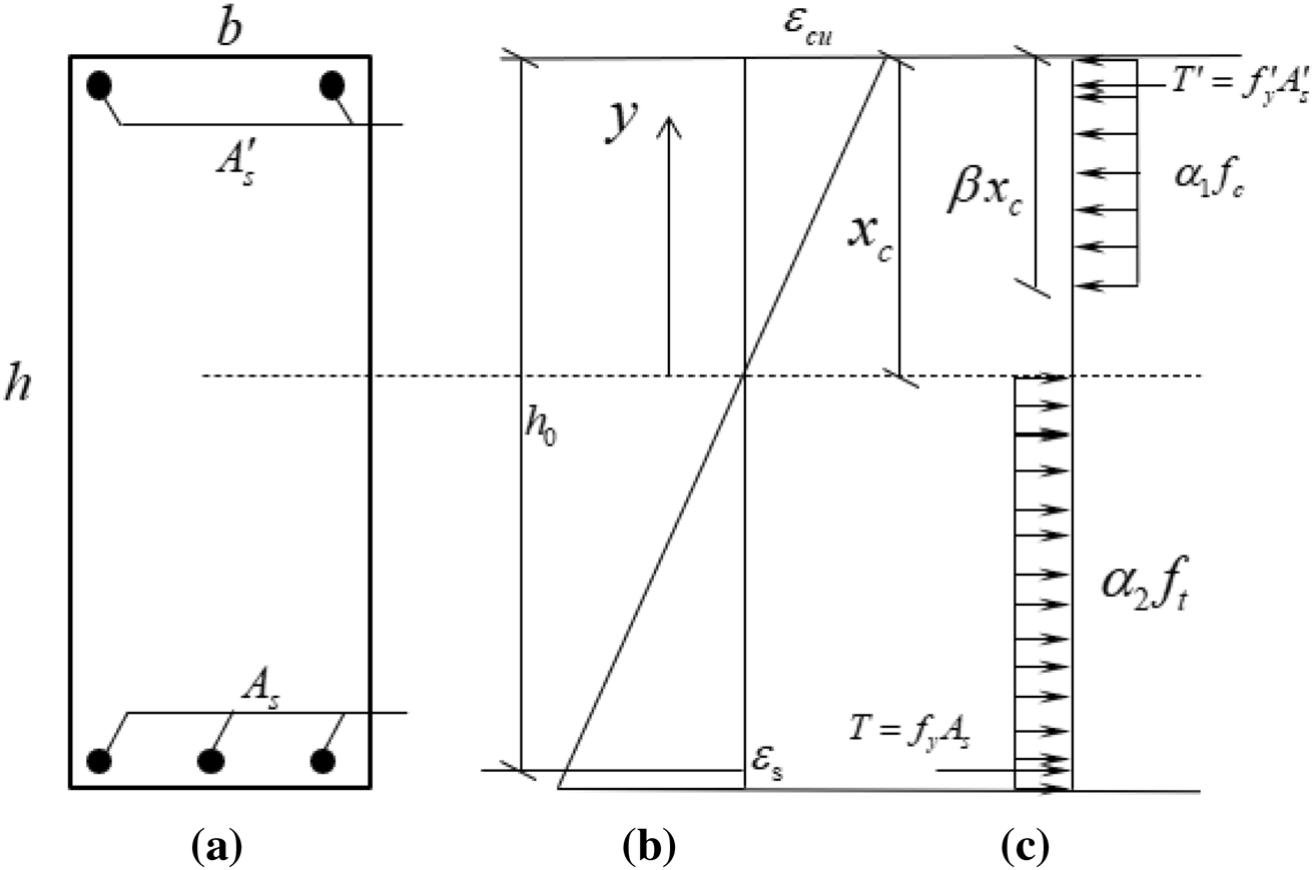
(**a**) Cross section. (**b**) Assumption of strain distribution at failure. (**c**) Equivalent of stress distribution at failure.

As shown in Fig. 9, the results of the compressive stress and tensile stress of concrete give:

3 $$ F_{c} = \alpha_{1} f_{c} b\beta x_{c} $$

4 $$ F_{t} = \alpha_{2} f_{t} b\left( {h - x_{c} } \right) $$

The moment of the compressive stress resultant about the neutral axis is

5 $$ M_{c} = \alpha_{1} f_{c} b\beta x_{c} \left( {x_{c} - \frac{1}{2}\beta x_{c} } \right) $$

Axial load equilibrium gives

6 $$ \left( {f_{y} A_{s} + \alpha_{2} f_{t} b\left( {h - x_{c} } \right)} \right) - \left( {f_{y}^{^{\prime}} A_{s}^{^{\prime}} + \alpha_{1} f_{c} b\beta x_{c} } \right) = 0 $$

where $$f_{y}$$ is the tensile yield strength of steel rebar, $$A_{s}$$ is the area of tensile longitudinal rebar, $$f_{y}^{^{\prime}}$$ is the compressive yield strength of steel rebar, $$A_{s}^{^{\prime}}$$ is the area of compressive longitudinal rebar, $$h$$ is the depth of the beam cross-section, $$b$$ is the width of the concrete section, $$x_{c}$$ is the distance from the top to the neutral axis,$$ f_{t}$$ is the tensile strength of the concrete, $$f_{c}$$ is the compressive strength of the concrete, $$\alpha_{1}$$, $$\alpha_{2}$$ and $$\beta$$ are the coefficients defining the depth and average stress of the equivalent rectangular stress block.

The ultimate moment can be evaluated as

7 $$ M_{u} = \left( {f_{y} A_{s} \left( {h_{0} - \frac{{\beta x_{c} }}{2}} \right) + f_{y}^{^{\prime}} A_{s}^{^{\prime}} \left( {\frac{{\beta x_{c} }}{2} - a_{s}^{^{\prime}} } \right) + \frac{1}{2}\alpha_{2} f_{t} b\left( {h - x_{c} } \right)\left( {h - \beta x_{c} + x_{c} } \right)} \right) $$

where $a_{s}^{^{\prime}}$ is the distance from the top to the compressive steel rebar and $h_{0}$ is the distance from the top to the tensile steel rebar.

Detailed properties of the beams are given in Table 6.

**Table 6:** Properties of the beam specimens.

Beam no	${f}_{y}$ (MPa)	${f}_{y}^{\mathrm{^{\prime}}}$ (MPa)	${A}_{s}$ (mm^2^)	${A}_{s}^{\mathrm{^{\prime}}}$ (mm^2^)	${f}_{t}$ (MPa)	$b$ (mm)	$h$ (mm)	${h}_{0}$ (mm)	${a}_{s}^{\mathrm{^{\prime}}}$ (mm)
NR	0	0	0	0	8.38	100	200	185	15
R12-1	360	300	227	101	8.38	100	200	184	16
R18-1	360	300	509	101	8.38	100	200	181	19
R18-2	360	300	1018	101	8.38	100	200	172	28
R20-2	360	300	1256	101	8.38	100	200	170	30
R22-2	360	300	1520	101	8.38	100	200	168	32

**Figure 10:**
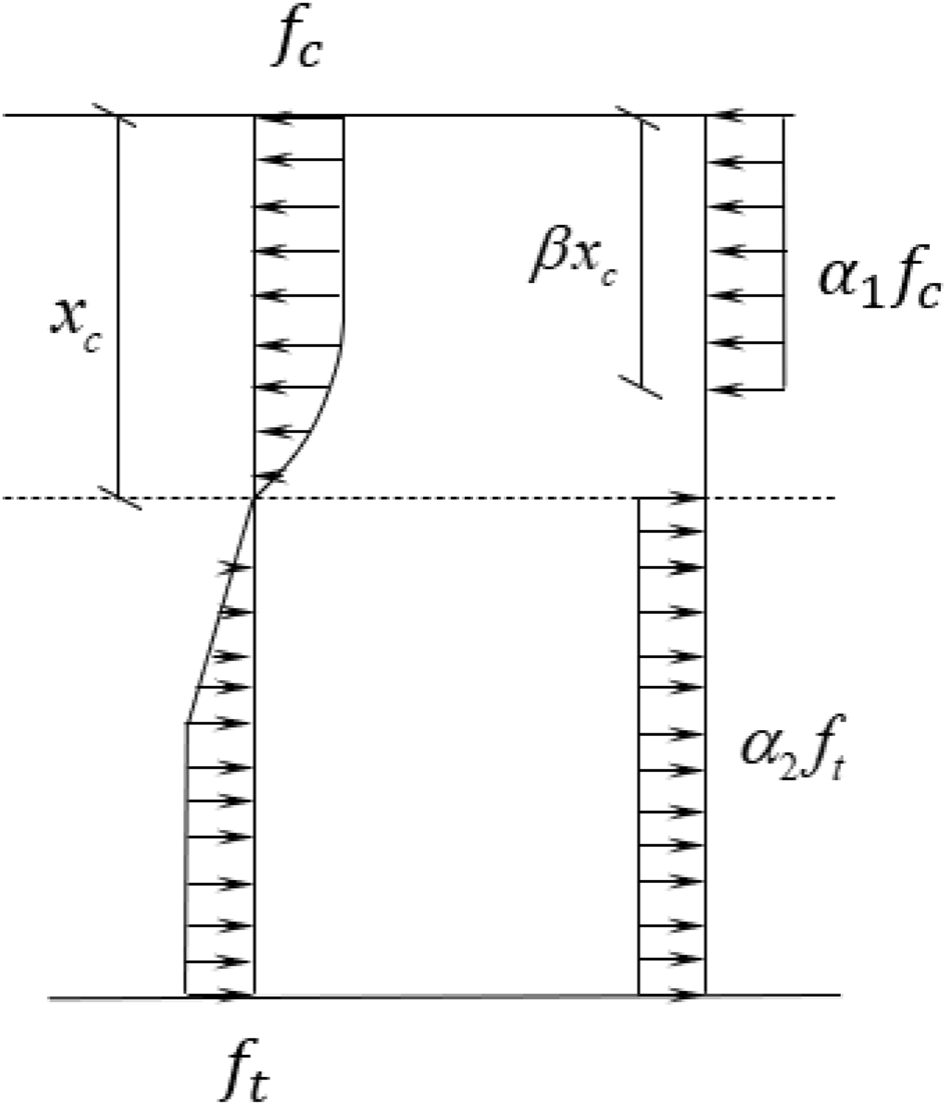
Equivalent of stress distribution.

To calculate the ultimate moment of the concrete beams, the parameters $\alpha_{2}$, $\beta$ and $x_{c}$ are needed to be determined. The equivalent of stress distribution at failure is from the assumption of stress distribution of cross-section at failure based on the constitutive relationship of UHPFRC at DBJ43T325-2017 as shown in Fig. 10.

The compressive stress and tensile stress resultant from the assumption of stress distribution at failure can be determined as follows.

8 $$ F_{c - assumption} = \mathop \smallint \limits_{0}^{{x_{c} }} \sigma_{c} \left( y \right)bdy $$

$$F_{t - assumption} = \mathop \smallint \limits_{0}^{{h - x_{c} }} \sigma_{t} \left( y \right)bdy$$ (9.

The moment of the compressive stress resultant about the neutral axis is

10 $$ M_{c - assumption} = \mathop \smallint \limits_{0}^{{x_{c} }} \sigma_{c} \left( y \right)bydy $$

where $\sigma_{c}$ is the compressive stress of concrete and $\sigma_{t}$ the tensile stress of the concrete.

The parameters $\alpha_{1}$ $\alpha_{2}$, $\beta$ and $x_{c}$ can be determined by combing equations of (3), (4), (5), (6), (8), (9), and (10) as follows.

11 $$ \left\{ {\begin{array}{*{20}c} {\alpha_{1} f_{c} b\beta x_{c} = \mathop \smallint \limits_{0}^{{x_{c} }} \sigma_{c} \left( y \right)bdy} \\ {\alpha_{2} f_{t} b\left( {h - x_{c} } \right) = \mathop \smallint \limits_{0}^{{h - x_{c} }} \sigma_{t} \left( y \right)bdy} \\ {\alpha_{1} f_{c} b\beta x_{c} \left( {x_{c} - \frac{1}{2}\beta x_{c} } \right){ } = \mathop \smallint \limits_{0}^{{x_{c} }} \sigma_{c} \left( y \right)bydy} \\ {\left( {f_{y} A_{s} + \alpha_{2} f_{t} b\left( {h - x_{c} } \right)} \right) - \left( {f_{y}^{^{\prime}} A_{s}^{^{\prime}} + \alpha_{1} f_{c} b\beta x_{c} } \right) = 0} \\ \end{array} } \right. $$

The value of parameters are summarized in Table 7. The details of calculating process are provided in "Appendix A".

**Table 7:** Calculation parameters.

${\alpha }_{1}$	${\alpha }_{2}$	$\beta$	${x}_{c}$(mm)					
			NR	R12-1	R18-1	R18-2	R20-2	R22-2
0.98	0.78	0.83	23.7	30.9	45.2	71.1	83.2	96.6

**Figure 11:**
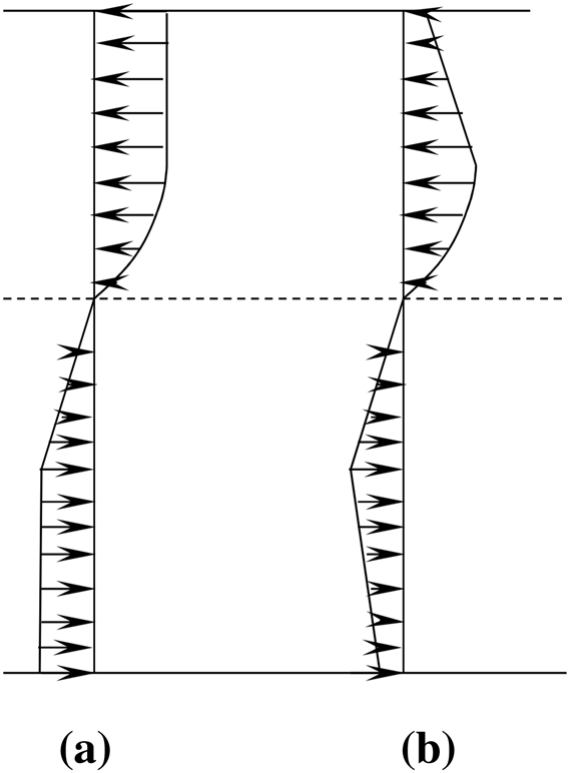
(**a**) Ideal equivalent of stress distribution in calculation model. (**b**) Possible equivalent stress distribution in the tests.

Table 8 gives the experimental and predicted bending moment capacity. It can be seen that the experimental bending moment capacity of the UHPFRC beams with reinforcement ratio from 0 to 7.85% were underestimated while that with reinforcement ratio of 9.50% were overestimated. The reason why the bending moment capacity was overestimated is that the stress distribution of concrete was based on ductile failure of concrete beams. The concrete crushed and the steel yielded simultaneously. However, as discussed in previous sections, the concrete was crushed without prior yielding of the steel for concrete beam of R22-2, leading to smaller stress distribution of concrete when the concrete beam was failure. Thus, this calculation model is not suitable for the beams with high reinforcement ratio. The stress distribution at DBJ43T325-2017 should be modified to a lower value. A possible stress distribution of concrete is shown in Fig. 11.

**Table 8:** Comparison of test results and prediction ($\eta$ represents relative error, Ave represents the mean value, and Stdev represents the standard deviation).

Beams	ρ (%)	Bending moment capacity ($kN\bullet m)$			$\eta$(%)
		Test results		Prediction	
		Ave	SD		
NR	0	10.65	1.21	9.97	6.41
R12-1	1.26	24.42	0.53	23.46	3.94
R18-1	2.83	44.28	2.56	38.97	11.99
R18-2	6.36	60.10	1.34	58.29	3.01
R20-2	7.85	66.95	3.92	66.48	0.70
R22-2	9.50	70.69	2.74	74.30	-5.10

## Conclusion

The properties of UHPFRC specimens with different dimensions curing at different temperature and the flexural behaviour of UHPFRC beams with reinforcement ratio from 0 to 9.5% curing at room temperature were investigated in the present study. Moreover, test results were used to compare with the theoretical results based on the numerical models from the Chinese standards. The following conclusions can be made.

The materials and mix proportions can be used to manufacture UHPFRC specimens with compressive strength over 150 MPa at curing temperature of 90 °C. The compressive strength of cubic UHPFRC specimens significantly decreased with the decrease of curing temperature from 90 to 20 °C. The cubic compressive strength of specimen with dimension of 100 × 100 × 100 mm^3^ cured at 20 °C and the prismatic compressive strength of specimen with dimension of 100 × 100 × 300 mm^3^ cured at 20 °C were 102.90 MPa and 79.08 MPa, respectively. The tensile strength and flexural strength of UHPFRC specimens cured at 20 °C were 8.38 MPa and 17.7 MPa, respectively.

The bearing capacity of UHPFRC beams cured at 20 °C under flexure was enhanced as the increase of reinforcement ratio. The failure modes of UHPFRC beams changed from ductile to brittle as the reinforcement ratio increased from 1.26 to 9.5%. Moreover, the ductility of UHPFRC beams decreased with the increase of reinforcement ratio. The effect of reinforcement ratio on the flexural behaviour of UHPFRC beams cured at room temperature exhibited similar effect on those cured at high temperature. However, the standard strength level of UHPFRC specimens cannot be achieved at curing temperature of 20 °C. Although there is some reduction in strength, the UHPFRC beams cured at room temperature showed good performance and the flexural behaviour of these beams were in accordance with the UHPFRC beams cured at high temperature.

Compared to the test results of the bending moment capacity of UHPFRC beams at room temperature, the calculation model of CECS38-2004 underestimated the bending moment capacity of the under-reinforced UHPFRC beams (with reinforcement ratio from 0 to 7.85%) and overestimated the bending moment capacity of the UHPFRC beams with high reinforcement ratio of 9.50%. Thus, the calculation model of CECS38-2004 can be used to estimate the bending moment capacity of UHPFRC with low reinforcement ratios cured at room temperature for the safe purpose of practical engineering.

## Supplementary Information

Supplementary Information.
