# Supplementary material for: Flexural behaviour and evaluation of ultra-high-performance fibre reinforced concrete beams cured at room temperature
Source: Sci Rep. 2021 Sep 24;11:19069. doi: 10.1038/s41598-021-98502-x (PMC8463697; doi:10.1038/s41598-021-98502-x)
Supplement: Supplementary file 1 — Supplementary Information. [file 41598_2021_98502_MOESM1_ESM.docx]

**Appendix A.**

The constitutive relationships of the UHPFRC from DBJ43T325-2017 are shown as followes.

$\sigma_{c}=f_{c}\left( 1-\left( 1-\frac{\varepsilon_{c}}{\varepsilon_{c0}} \right)^{n} \right)$ $0\leq\varepsilon_{c}\leq\varepsilon_{c0}$ (A-1)

$n=2-\frac{1}{60}(f_{cu,k}-50)$ (A-2)

$\sigma_{c}=f_{c}=const$ $\varepsilon_{c0}\leq\varepsilon_{c}\leq\varepsilon_{cu}$ (A-3)

$\sigma_{t}=f_{t}\frac{\varepsilon_{t}}{\varepsilon_{t0}}$ $0\leq\varepsilon_{t}\leq\varepsilon_{t0}$ (A-4)

$\sigma_{t}=f_{t}=const$ $\varepsilon_{t0}\leq\varepsilon_{t}\leq\varepsilon_{tu}$ (A-5)

where $\sigma_{c}$ is the compressive stress of concrete, $f_{c}$ is the compressive strength of concrete, $f_{cu,k}$ is the compressive strength of specimen with dimension of 150 × 150 × 150 mm^3^, $\sigma_{t}$ the tensile stress of the concrete, $f_{t}$ is the tensile strength of the concrete, $\varepsilon_{c}$ is the compressive strain of the concrete, $\varepsilon_{c0}$ is the compressive strain when compressive stress reaches up to $f_{c}$, $\varepsilon_{t}$ is the tensile strain of the concrete, $\varepsilon_{t0}$ is the tensile strain when tensile stress reaches up to $f_{t}$ $\varepsilon_{cu}$ is the ultimate compressive strain of the concrete and $\varepsilon_{tu}$ is the

Figure A-1 shows the stress-strain response of concrete with steel fibres according to equation A-1 to A-5.

Fig A-1. Stress-strain response of concrete with steel fibre.

Thus, from Fig 10 and Fig A-1, the equation set (9) can be rewritten as follow.

$\begin{aligned} \alpha_{1}f_{c} b\beta x_{c}=\int_{0}^{\frac{\varepsilon_{c0}}{\varepsilon_{cu}}x_{c}} f_{c}\left( 1-\left( 1-\frac{\varepsilon_{c}}{\varepsilon_{c0}} \right)^{n} \right)bdy+\int_{\frac{\varepsilon_{c0}}{\varepsilon_{cu}}x_{c}}^{x_{c}} f_{c}bdy \\ {\alpha_{2}f}_{t}b\left( h-x_{c} \right)=\int_{0}^{\frac{\varepsilon_{t0}}{\varepsilon_{tu}}(h-x_{c})} f_{t}\frac{\varepsilon_{t}}{\varepsilon_{t0}}bdy+\int_{\frac{\varepsilon_{t0}}{\varepsilon_{tu}}(h-x_{c})}^{h-x_{c}} f_{t}bdy \\ \alpha_{1}f_{c} b\beta x_{c}\left( x_{c}-\frac{1}{2}\beta x_{c} \right) =\int_{0}^{\frac{\varepsilon_{c0}}{\varepsilon_{cu}}x_{c}} f_{c}\left( 1-\left( 1-\frac{\varepsilon_{c}}{\varepsilon_{c0}} \right)^{n} \right)bydy+\int_{\frac{\varepsilon_{c0}}{\varepsilon_{cu}}x_{c}}^{x_{c}} f_{c}bydy \\ \left( f_{y}A_{s}+{\alpha_{2}f}_{t}b\left( h-x_{c} \right) \right)-\left( f_{y}^{'}A_{s}^{'}+\alpha_{1}f_{c} b\beta x_{c} \right)=0 \end{aligned}$ (A-6)

According to Fig 10, the relationship of the compressive strain of concrete and the depth is as follows.

$\varepsilon_{c}=\varepsilon_{cu}\frac{y}{x_{c}}$ (A-7)

$\varepsilon_{t}=\varepsilon_{s}\frac{y}{h-x_{c}}$ (A-8)

Table A-1 gives the properties of the concrete beams which have been determined in the main body of this study. To solve the equation set (9), the value of $\varepsilon_{c0}$, $\varepsilon_{t0}$, $\varepsilon_{cu}$, $\varepsilon_{tu}$ and $n$ are determined as follows.

The cube compressive strength obtained in this study is from specimens with dimension of 100 × 100 × 100 mm^3^ while the dimension for the value of $f_{cu,k}$ is 150 × 150 × 150 mm^3^. According to GB50081-2010, $f_{cu,k}$ can be 0.95 times of the cube compressive strength of specimens with dimension of 100 × 100 × 100 mm^3^.

Thus, the value of $f_{cu,k}$ can be obtained as follow.

$f_{cu,k}=0.95\times102.90=97.96 MPa$

$n=2-\frac{1}{60}\left( f_{cu,k}-50 \right)=1.2$

The value of $\varepsilon_{c0}$ and $\varepsilon_{cu}$ can be obtained from the compression test of prismatic specimens. As shown in Fig A-2, $\varepsilon_{c0}=2600 \mu\varepsilon$ and $\varepsilon_{cu}=6400 \mu\varepsilon$ in this study.

The value of $\varepsilon_{t0}$ and $\varepsilon_{tu}$ can be obtained from tension tests of dog-bone shaped specimen. As shown in Fig A-3, $\varepsilon_{t0}=1923 \mu\varepsilon$ and $\varepsilon_{tu}=3142 \mu\varepsilon$ in this study.

Table A-2 lists the value of $\varepsilon_{c0}$, $\varepsilon_{cu}$, $\varepsilon_{t0}$, $\varepsilon_{tu}$ and $n$.

According to Table A-1 and A-2, the value of $\alpha_{1}$, $\alpha_{2}$, $\beta$ and $x_{c}$ can be obtained by substituting the parameters in equation set (9). Table A-3 shows the value of $\alpha_{1}$, $\alpha_{2}$, $\beta$ and $x_{c}$ for all the concrete beams.

Table A-1. Properties of the concrete beams.

| Beam no. | | NR | R12-1 | R18-1 | R18-2 | R20-2 | R22-2 |
| --- | --- | --- | --- | --- | --- | --- | --- |
| $f_{y}$ (MPa) | | 0 | 360 | 360 | 360 | 360 | 360 |
| $f_{y}^{'}$ (MPa) | | 0 | 300 | 300 | 300 | 300 | 300 |
| $A_{s}$ (mm^2^) | | 0 | 227 | 509 | 1018 | 1256 | 1520 |
| $A_{s}^{'}$ (mm^2^) | | 0 | 101 | 101 | 101 | 101 | 101 |
| $f_{t}$ (MPa) | | 8.38 | 8.38 | 8.38 | 8.38 | 8.38 | 8.38 |
| $f_{c}$ (MPa) | 100 × 100 × 100 (mm^3^) | 102.90 | 102.90 | 102.90 | 102.90 | 102.90 | 102.90 |
|  | 100 × 100 ×300 (mm^3^) | 79.08 | 79.08 | 79.08 | 79.08 | 79.08 | 79.08 |
| $b$ (mm) | | 100 | 100 | 100 | 100 | 100 | 100 |
| $h$ (mm) | | 200 | 200 | 200 | 200 | 200 | 200 |
| $h_{0}$ (mm) | | 185 | 184 | 181 | 172 | 170 | 168 |
| $a_{s}^{'}$ (mm) | | 15 | 16 | 19 | 28 | 30 | 32 |

Table A-2. Value of UHPFRC parameters

| Parameters | $\varepsilon_{c0}$ | $\varepsilon_{t0}$ | $\varepsilon_{cu}$ | $\varepsilon_{tu}$ | $n$ |
| --- | --- | --- | --- | --- | --- |
| Value | 2600με | 1923με | 6400με | 3142με | 1.2 |

Table A-3. The value of parameters for concrete beams

| $\alpha_{1}$ | $\alpha_{2}$ | $\beta$ | $x_{c}$ (mm) | | | | | |
| --- | --- | --- | --- | --- | --- | --- | --- | --- |
|  |  |  | NR | R12-1 | R18-1 | R18-2 | R20-2 | R22-2 |
| 0.98 | 0.78 | 0.83 | 23.7 | 30.9 | 45.2 | 71.1 | 83.2 | 96.6 |

Fig A-2. Compression test result of prismatic specimen

Fig A-3. Tension test results of dog-bone shaped specimen
